# Supplementary material for: Immune Cell Density Evaluation Improves the Prognostic Values of Staging and p16 in Oropharyngeal Cancer
Source: Cancers (Basel). 2022 Nov 12;14(22):5560. doi: 10.3390/cancers14225560 (PMC9688704; doi:10.3390/cancers14225560)
Supplement: Supplementary file 1 [file cancers-14-05560-s001.zip › cancers-2008091-supplementary.pdf]

Supplementary Table S1. Description of immunostaining experimental conditions.

**Immunostaining**	**CD8**	**FoxP3**	**CD68**	**CD1a**
Antigen retrieval				
Buffers	EDTA 10%	Citrate 10%	EDTA 10%	EDTA 10%
Conditions	*PC 6 min	*MW 21 min	PC 6 min	PC 11 min
Blocking	Casein 0.5% 15 min	CSAII kit	Casein 0.5% 15 min	Casein 0.5% 1 h
Primary antibodies				
Species	Mouse	Mouse	Mouse	Mouse
Source	Dako	Invitrogen	Dako	Dako
Dilution	1/200	1/200	1/200	1/20
Timing	1 h *RT	1 h RT	1 h RT	Overnight 4°C
Secondary antibodies				
Kit	BrightVision Poly- HRP-Anti Mouse/Rabbit IgG, VWR	CSAII kit, Dako	BrightVision Poly- HRP-Anti Mouse/Rabbit IgG, VWR	BrightVision Poly- HRP-Anti Mouse/Rabbit IgG, VWR

*PC = Pressure Cooker, MW = Micro-Waves, RT = Room Temperature
